# JIP1 Deficiency Protects Retinal Ganglion Cells From Apoptosis in a Rotenone-Induced Injury Model

**DOI:** 10.3389/fcell.2019.00225

**Published:** 2019-10-15

**Authors:** Wenyi Liu, Xue Li, Xi Chen, Jieqiong Zhang, Linlin Luo, Qiumei Hu, Jiaxing Zhou, Jun Yan, Sen Lin, Jian Ye

**Affiliations:** ^1^Department of Ophthalmology, Daping Hospital, Army Medical Center of PLA, Army Medical University, Chongqing, China; ^2^Department 1, Research Institute of Surgery & Daping Hospital, Army Medical University, Chongqing, China

**Keywords:** JIP1, RGC, apoptosis, JNK, LHON

## Abstract

Retinal ganglion cells (RGCs) undergo apoptosis after injury. c-Jun N-terminal kinase (JNK)-interacting protein 1 (JIP1) is a scaffold protein that is relevant to JNK activation and a key molecule known to regulate neuronal apoptosis. However, the specific role of JIP1 in the apoptosis of RGCs is currently undefined. Here, we used JIP1 gene knockout (KO) mice to investigate the importance of JIP1-JNK signaling in the apoptosis of RGCs in a rotenone-induced injury model. In adult JIP1 KO mice, the number and electrophysiological functions of RGCs were not different from those of wild-type (WT) mice. Ablation of JIP1 attenuated the activation of JNK and the cleavage of caspase-3 in the retina after rotenone injury and contributed to a lower number of TUNEL-positive RGCs, a greater percentage of surviving RGCs, and a significant reduction in the electrophysiological functional loss of RGCs when compared to those in WT controls. We also found that JIP1 was located in the neurites of primary RGCs, but accumulated in soma in response to rotenone treatment. Moreover, the number of TUNEL-positive RGCs, the level of activation of JNK and the rate of cleavage of caspase-3 were reduced in primary JIP1-deficient RGCs after rotenone injury than in WT controls. Together, our results demonstrate that the JIP1-mediated activation of JNK contributes to the apoptosis of RGCs in a rotenone-induced injury model *in vitro* and *in vivo*, suggesting that JIP1 may be a potential therapeutic target for RGC degeneration.

## Introduction

Retinal ganglion cell (RGC) impairment is a common event, in a variety of ocular diseases. The pathologic changes of RGCs induced by intravitreal injection of rotenone to mice may resemble the mitochondria-related RGC impairment observed in Leber hereditary optic neuropathy (LHON) patients (Zhang et al., 2002, 2006; de la Barca et al., 2016). LHON is one of the major causes of selective RGC death in the retina and results in the impairment of visual signal propagation and subsequent progressive visual field defects in patients (Yu-Wai-Man et al., 2011). It is the most common mitochondrial disorder, with few treatments currently available (Yu-Wai-Man et al., 2009). Therapy for the prevention of LHON is receiving increasing attention.

The c-Jun N-terminal kinase (JNK) pathway is a known critical regulator of neuronal death in many diseases such as Alzheimer’s disease (AD) (Ando et al., 2011; Wang et al., 2013), Parkinson’s disease (PD) (Huang et al., 2016; Spigolon et al., 2018) and glaucoma (Welsbie et al., 2013; Mammone et al., 2018), and is considered a potential target for neuroprotective therapy (Wang et al., 2012; Kim et al., 2016). JNKs are involved in the extrinsic apoptotic pathway initiated by death receptors as well as in the intrinsic pathway initiated at the mitochondrial level. Reactive oxygen species (ROS) triggered JNK and sterol regulatory element binding protein (SREBP) activity in neurons leads to LD accumulation, which is related to neurodegeneration, and this process can be delayed by blocking JNK (Liu et al., 2015). JNK interacts with mitochondrial Sab, leading to impaired respiration and increased apoptosis, suggesting that blocking JNK activation contributes to its anti-apoptotic effects (Win et al., 2014). Therefore, mitochondrial dysfunction-related neuronal degeneration and impairment can be suppressed or closely related by inhibiting JNK signaling. However, whether blocking JNK exerts a neuroprotective effect in LHON remains to be elucidated.

c-Jun N-terminal kinase (JNK)-interacting protein 1 (JIP1) is a scaffold protein in the JNK signaling pathway (Morrison and Davis, 2003). Previous studies have suggested that JIP1 can assemble a functional JNK activation module composed of several bound MAP3Ks as well as MKK7, and JNK (Whitmarsh et al., 1998). This complex may be relevant to the JNK activation that is caused by the exposure of cells to multiple forms of stress (Kennedy et al., 2007; Morel et al., 2010; Xu et al., 2010). Whitmarsh et al. reported that JIP1-deficient mice were viable and had a normal life span. The authors also indicated that the JIP1 null mutation prevented stress-induced JNK activation and hippocampal neuronal cell death *in vivo* and *in vitro* (Whitmarsh et al., 2001). The visual system, including RGCs and other cells, is a part of the central nervous system and is influenced by JNK activation in several pathologic conditions (Bessero et al., 2010; Fernandes et al., 2012). However, detrimental signaling molecules and mechanistic pathways associated with mitochondrial dysfunction-related retinal degeneration and its relationship with JIP1 remain unclear.

In this study, we evaluated the role of the JIP1-JNK signaling pathway in retinal degeneration in both retinal cell culture and a mouse retinal rotenone injury model. First, we examined the effect of JIP1 deficiency on rotenone-induced changes in the structure and function of the mouse retina. Second, we examined the regulation of cell death by JIP1 in primary RGCs. Finally, we examined molecular signaling alterations in JIP1-deficient mice. These data show that the JIP1-JNK signaling pathway is critical for rotenone-induced RGC death and that JIP1 deficiency prevents neuronal death and exerts morphological and functional protective effects on RGCs in rotenone injury models.

## Results

To study the function of JIP1 in RGCs, we generated a strain of JIP1 KO mice. In this strain, exon 3, which encodes the JNK-binding domain (JBD) of the JIP1 gene, was replaced with a neomycin resistance cassette. The homozygous mice are viable, fertile and normal in size, as described by Whitmarsh et al. (2001). The analysis of retinas showed that the JIP1 mRNA and protein were not detected in JIP1 KO mice (Supplementary Figure S1).

### JIP1 Deficiency Protects RGCs From Loss After Rotenone-Induced Injury

**FIGURE 1:**
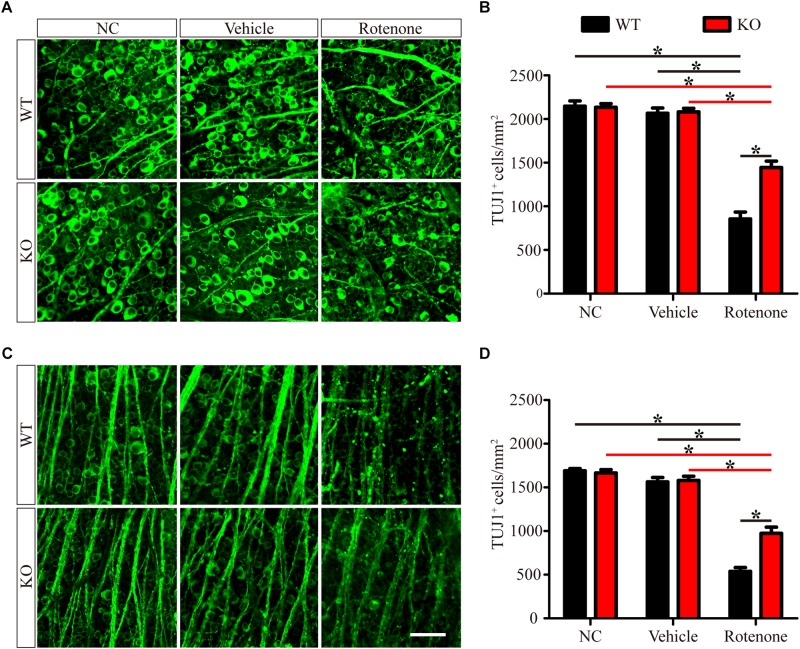
JIP1 deficiency protects against RGC soma loss after rotenone treatment. RGCs were immunostained using a TUJ1 antibody in the distal **(A)** and proximal **(C)** regions of retinal wholemounts from WT and JIP1 KO mice subjected to different treatments. Quantification of TUJ1-positive cells in the distal **(B)** and proximal **(D)** regions (^∗^*P* < 0.05, *n* = 12 images, scale bar = 50 μm). NC, negative control.

To determine the detrimental or beneficial roles of JIP1 in RGC survival, we injected rotenone into the vitreous bodies of WT and JIP1 KO mice. RGCs were immunostained with a TUJ1 antibody and counted in the distal (Figure 1A) and proximal (Figure 1C) regions of the retinal wholemounts. In both the distal and proximal regions, WT and JIP1 KO mice had similar numbers of RGCs (Figures 1B,D), indicating that the number of RGCs in the retinas of these mice was not significantly influenced by JIP1 deficiency. The same amount of vehicle was injected via the same method used for rotenone treatment to rule out the effect of the solvent. The number of RGCs in both regions was not significantly different between the vehicle-treated retinas and the untreated groups with the same genotypes (Figures 1B,D). These data indicate that the vehicle did not influence the RGC number. Moreover, after intravitreal injection of rotenone, the JIP1-deficient mice had a larger number of surviving RGCs than were found in the WT mice (Figures 1B,D). These data demonstrate that JIP1 deficiency protected RGC somas from loss in a rotenone-induced RGC death model.

### JIP1 Deficiency Protects the Ganglion Cell Complex (GCC) From Thinning After Rotenone-Induced Injury

Spectral-domain optical coherence tomography (SD-OCT) is an ideal method for performing clinical and experimental retinal examinations without labeling *in vivo* (Morgan et al., 2017). The resolution of SD-OCT allows the detection of cellular organelles, and the loss of RGC dendrites and fragmentation as a result of axonal damage can alter the light-scattering behavior of degenerating neurons and can be used to evaluate quantitative measurements of RGC damage (Nouri-Mahdavi et al., 2013; Blanch et al., 2018).

**FIGURE 2:**
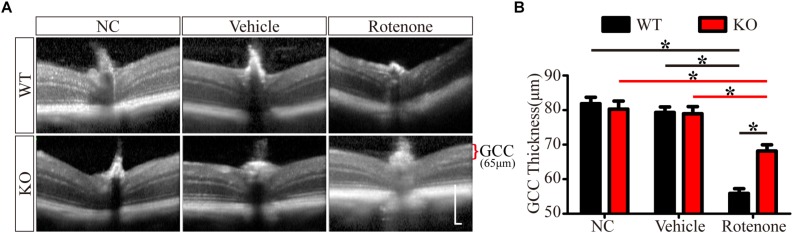
JIP1 deficiency protects the GCC from thinning. **(A)** OCT scanning results of the retinas of WT and JIP1 KO mice subjected to different treatments (scale bar = 200 μm). **(B)** Quantification of the GCC thickness in WT and JIP1 KO mice subjected to different treatments (^∗^*P* < 0.05, *n* = 6).

Enlarged images of retinal SD-OCT scans of WT and JIP1 KO mice subjected to different treatments are shown in Figure 2A. We investigated the thickness of the GCC, which is a combination of the retinal nerve fiber layer (RNFL), ganglion cell layer (GCL) and inner plexiform layer (IPL) and is useful for evaluating the structural changes in RGC degeneration diseases (Kim N.R. et al., 2010; Kotowski et al., 2012; Ohkubo et al., 2014). The GCC thicknesses of the WT and JIP1 KO mice were not significantly different. Similarly, the thickness of the GCC was not significantly different between groups after vehicle injection. In addition, GCC was significantly thicker in the JIP1 KO mice than in the WT mice after intravitreal injection of rotenone (Figure 2B). These data demonstrate that JIP1 deficiency attenuates rotenone-induced GCC thinning.

### JIP1 Deficiency Protects the Function of RGCs and Their Axons After Rotenone-Induced Injury

The photopic negative response (PhNR) of the light-adapted (LA) electroretinogram (ERG) is a negative-going wave that occurs after the b-wave and provides information about the function of RGCs and their axons (Frishman et al., 2018). The amplitude of this wave can be reduced in the early stages of disorders that affect the innermost retina, including glaucoma and other forms of optic neuropathy (Kizawa et al., 2006; Miyata et al., 2007; Preiser et al., 2013). This wave can be recorded in mice and provides a means for evaluating the functions of RGCs and their axons in mouse models (Chrysostomou and Crowston, 2013).

**FIGURE 3:**
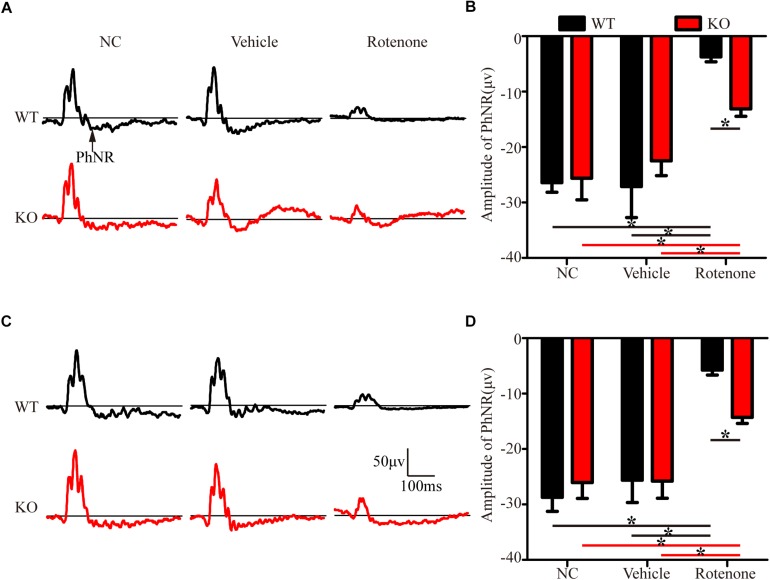
JIP1 deficiency protects the function of RGCs and their axons. Representative traces demonstrating the PhNR components (arrows) that were recorded from a mouse with a stimulus strength of 19.49 cd.s/m^2^ **(A)** and 41.68 cd.s/m^2^ **(C)**. Quantification of PhNR amplitudes with a stimulus strength of 19.49 cd.s/m^2^ **(B)** and 41.68 cd.s/m^2^ **(D)** (^∗^*P* < 0.05, *n* = 6).

In the present study, photopic ERG responses were recorded under two different stimulus strengths in individual mice before and at 24 h after the intravitreal injection of vehicle or rotenone. Figures 3A,C show representative ERG waves with stimulus strengths of 19.49 and 41.68 cd.s/m^2^, respectively. The amplitudes of the PhNR of the WT, JIP1 KO, WT + vehicle, and JIP1 KO + vehicle groups were not significantly different for either stimulus strength (Figures 3B,D). While rotenone treatment significantly reduced the amplitude of the PhNR in WT mice, the amplitude of the PhNR was significantly higher in JIP1 KO mice than in WT mice with the same rotenone treatment (Figures 3B,D). These results suggest that JIP1 deficiency attenuated the decrease in the PhNR amplitude and may protect the electrophysiological function of RGCs and their axons after rotenone treatment.

### JIP1 Deficiency Protects RGCs From Apoptosis After Rotenone-Induced Injury

**FIGURE 4:**
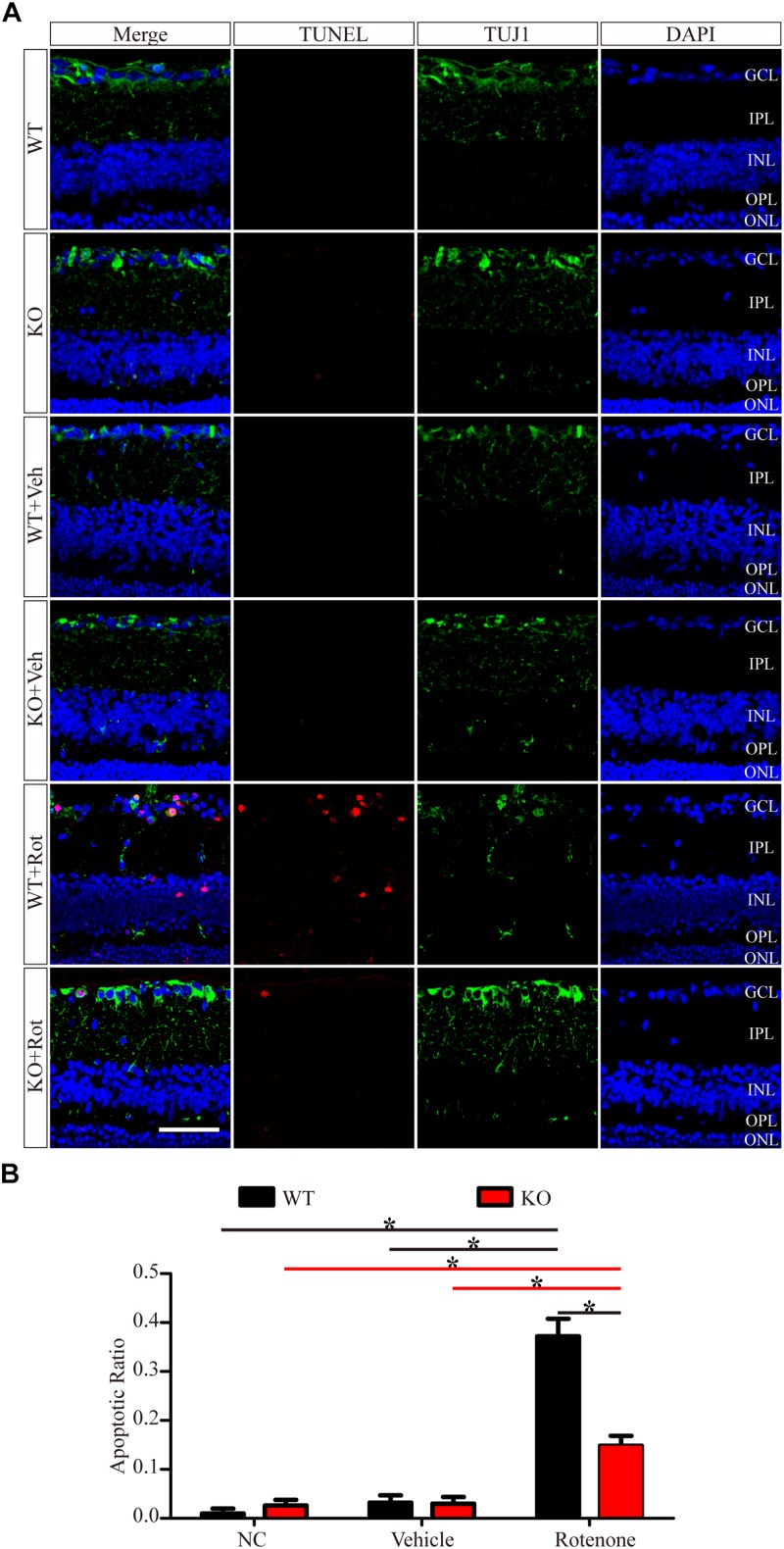
JIP1 deficiency protects RGCs from apoptosis *after rotenone-induced* injury. **(A)** TUNEL (red), TUJ1 (green) and DAPI (blue) staining show apoptotic cells and RGCs in retinal sections of different groups (scale bar = 20 μm). **(B)** Quantification of TUNEL-positive cells vs. DAPI-positive cells, which is shown as an apoptotic ratio (^∗^*P* < 0.05, *n* = 6). GCL, ganglion cell layer; IPL, inner plexiform layer; INL, inner nuclear layer; OPL, outer plexiform layer; ONL, outer nuclear layer; NC, negative control; Veh, vehicle; Rot, rotenone.

To identify apoptosis, retinas were assessed using the TUNEL technique. As shown in Figure 4A, the TUNEL-positive cells were mainly localized in the GCL. A few TUNEL-positive cells were detected in control eyes without intravitreal injection and in vehicle-treated eyes in both WT and JIP1 KO mice. Apoptosis was significantly induced by the intravitreal injection of rotenone in the WT group, while fewer TUNEL-positive cells were observed in the GCL in the JIP1 KO group (Figures 4A,B). These data indicate that JIP1 deficiency protects RGCs from apoptosis after rotenone-induced injury. However, the cellular and molecular mechanisms need to be further explored.

### Rotenone Triggers the Translocation of Jip1 in Rgcs

**FIGURE 5:**
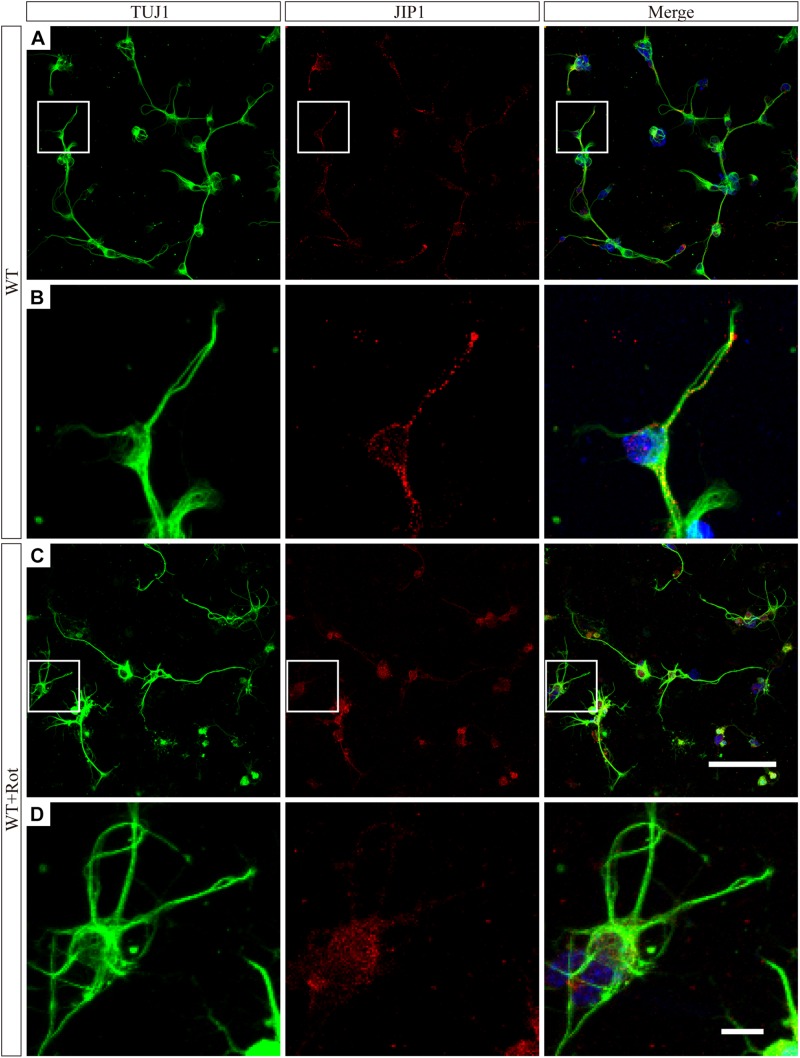
JIP1 translocats to the cytoplasm following rotenone treatment. JIP1 (red), TUJ1 (green) and DAPI (blue) were examined in primary RGCs isolated from WT mice without **(A)** or with **(C)** rotenone treatment at low magnification (scale bar = 100 μm). **(B)** Magnified images of the square areas in panel **(A)**. **(D)** Magnified images of the square areas in panel **(C)** (**B,D**, scale bar = 10 μm). The TUJ1 antibody reacts with beta-tubulin III, and is widely used as a marker to distinguish RGCs in retinas.

We prepared primary RGCs from WT pups to investigate the subcellular distribution of JIP1. Immunofluorescence analysis using an antibody against JIP1 showed that RGCs had diffuse JIP1 staining in the cytoplasm, including soma and the tips of extended neurites. In comparison, following rotenone treatment, most of the JIP1 was detected in the soma and the area surrounding the nuclei (Figure 5). This was similar to the distribution of JIP1 in the primary cultures of cortical neurons (Whitmarsh et al., 2001). These data demonstrate the expression pattern of JIP1 in RGCs and suggest that the subcellular localization of JIP1 was altered in RGCs following exposure to cellular mitochondrial stress and that this change may be related to the biological activity of downstream molecules. However, the molecular mechanism by which JIP1 contributes to RGC protection requires further investigation.

### JIP1 Deficiency Represses JNK Activation to Protect RGCs From Apoptosis

JIP1 plays an important role in the regulation of JNK activity, as shown in several *in vitro* and *in vivo* studies (Jaeschke et al., 2004; Kim M.Y. et al., 2010; Hein et al., 2016; Morel et al., 2018). JNK is activated via the phosphorylation of threonine and tyrosine residues, and several types of injuries may activate JNK and result in various biological outcomes (Jin et al., 2018; Levada et al., 2018; Zhang et al., 2018). Therefore, we assessed pJNK immunoreactivity to determine whether the protection to RGCs during rotenone-induced injury that results from JIP1 deficiency is associated with JNK phosphorylation.

**FIGURE 6:**
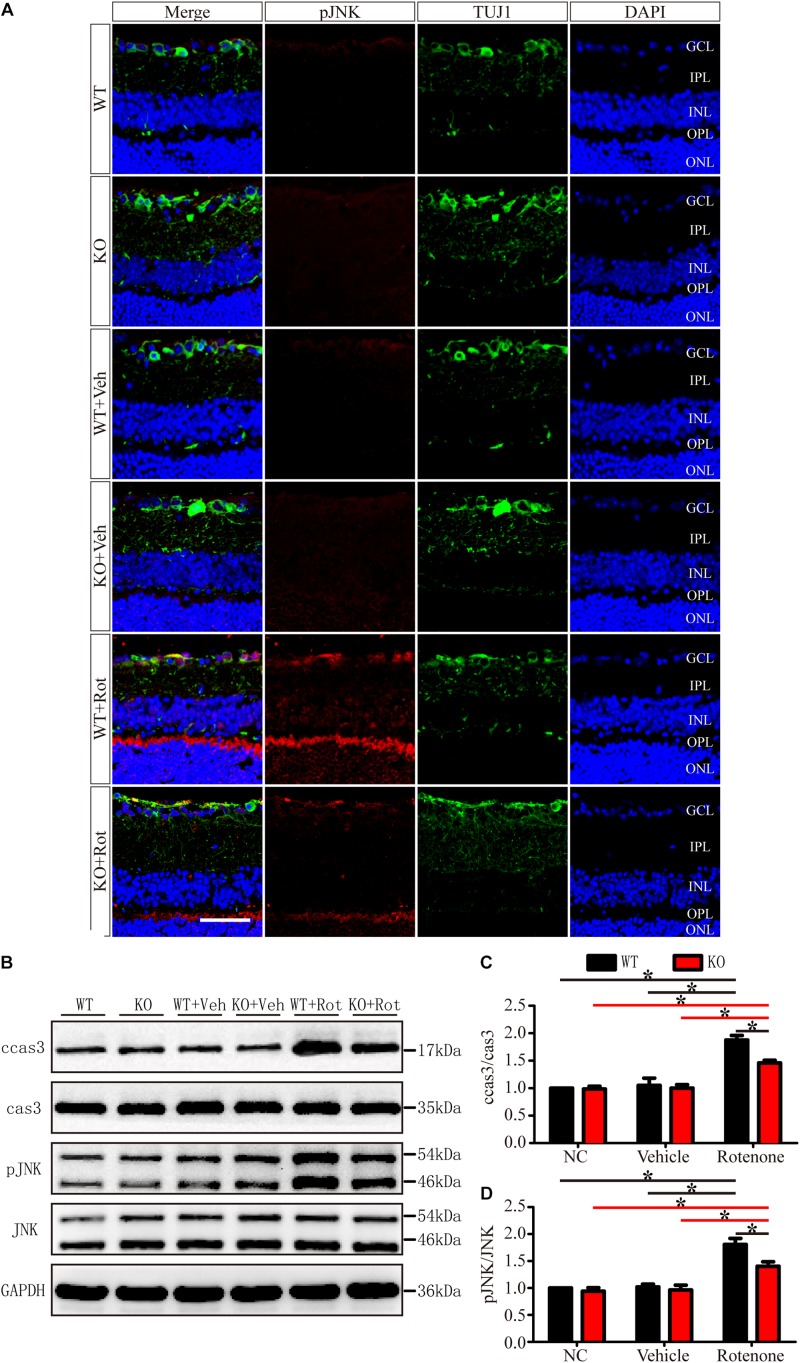
JIP1 deficiency represses JNK phosphorylation and cleaved caspase-3 signaling in a rotenone-induced injury model. **(A)** Immunostaining of pJNK (red), TUJ1 (green) and DAPI (blue), showing JNK activity in retinal sections from different groups (scale bar = 50 μm). **(B)** Representative images of Western blot results of ccas3, cas3, pJNK, JNK and the loading control GAPDH in retinas. **(C)** Quantification of the Western blot results for the ratios of cleaved caspase-3 to caspase-3 in the different groups (^∗^*P* < 0.05, *n* = 3). **(D)** Quantification of the Western blot results for the ratios of pJNK to JNK in the different groups (^∗^*P* < 0.05, *n* = 3). GCL, ganglion cell layer; IPL, inner plexiform layer; INL, inner nuclear layer; OPL, outer plexiform layer; ONL, outer nuclear layer; ccas3, cleaved caspase-3; cas3, caspase-3; NC, negative control; Veh, vehicle; Rot, rotenone.

As shown in Figure 6, in the normal retinas of JIP1 KO mice, JNK activity, determined by the phosphor-JNK level, was not significantly different from that observed in the retinas of WT mice (Figures 6A,B,D), indicating that the basal activity of JNK in the retinas of the mice we used in this study was maintained in the absence of JIP1. We further tested whether JIP1 activates JNK in a rotenone-induced injury model. After the intravitreal injection of rotenone, the levels of phospho-JNK in the retina were notably higher than those in the non-treated groups and the vehicle-treated groups in both WT and JIP1 KO mice. The levels of phospho-JNK in JIP1 KO mice were significantly lower than those in WT mice (Figures 6A,B,D).

Cleaved caspase-3 is a classic apoptotic marker for mitochondrial apoptosis in RGCs. After intravitreal injection of rotenone, the ratio of cleaved caspase-3 to caspase-3 was clearly higher in the WT group than in the non-treated group, vehicle-treated group and JIP1 KO rotenone-treated group (Figures 6B,C).

These data indicate that JIP1 deficiency protected RGCs from apoptosis, and that this effect depend on the repression of JNK activation and the further regulation of cleaved caspase-3/caspase-3 signaling after rotenone-induced injury.

**FIGURE 7:**
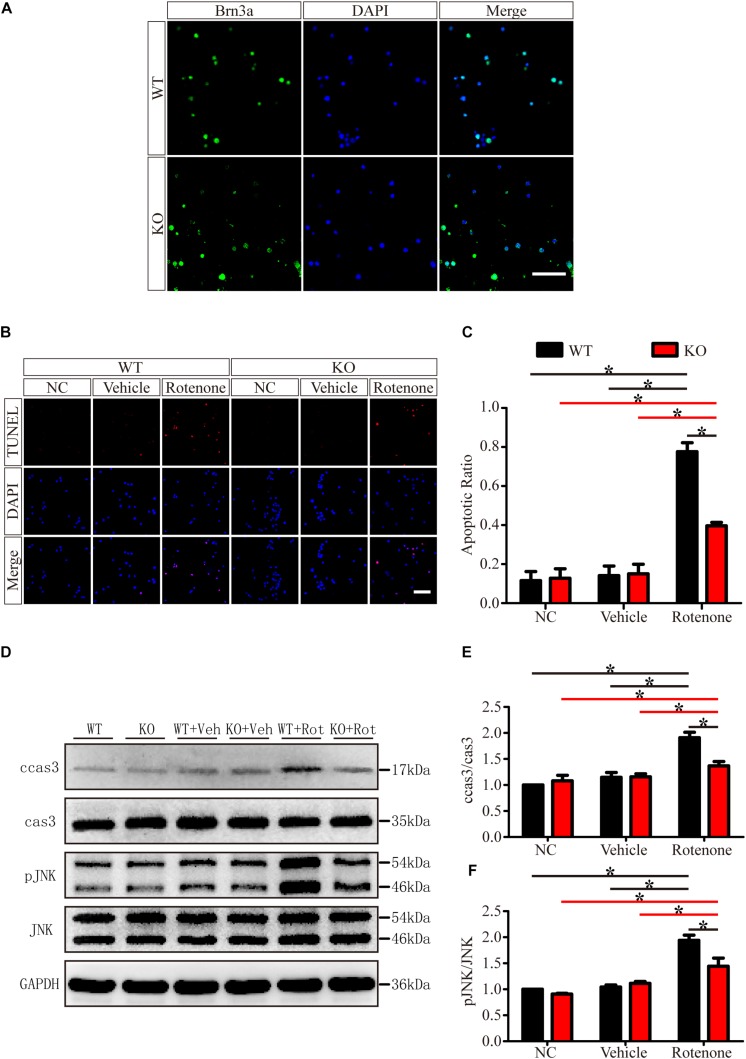
JIP1 deficiency protects primary RGCs from apoptosis after rotenone-induced injury. **(A)** Identification of primary RGCs using Brn3a immunostaining. **(B)** TUNEL (red) staining was used to analyze the apoptotic cells in the primary RGCs of different groups (scale bar = 20 μm). **(C)** Quantification of the ratio of TUNEL-positive cells and DAPI-positive cells, which is shown as the TUNEL ratio (^∗^*P* < 0.05, *n* = 6). **(D)** Representative images of the Western blot results of ccas3, cas3, pJNK, JNK and the loading control GAPDH in the different groups of primary RGCs. **(E,F)** Quantification of Western blot results showing the ratios of ccas3 to cas3 and pJNK to JNK in the different groups of primary RGCs (^∗^*P* < 0.05, *n* = 3). NC, negative control; Veh, vehicle; Rot, rotenone.

To exclude the cellular non-autonomous phenotype and confirm the *in vivo* results, we cultured and purified primary RGCs from WT and JIP1 KO mice. Primary RGCs were identified using the specific marker Brn3a, and as shown in Figure 7A, almost all of the cultured cells were RGCs. After 12 h of rotenone treatment, there were more TUNEL-positive cells in the WT group than in the JIP1 KO group, and the apoptotic ratios in the WT, JIP1 KO, WT + vehicle and JIP1 KO + vehicle groups were not significantly different (Figures 7B,C). Western blot results showed that the levels of cleaved caspase-3 and pJNK were higher in WT RGCs treated with rotenone than in JIP1 KO RGCs with the same treatment, which was consistent with the *in vivo* results (Figures 7D,E). These data confirm that JIP1 deficiency protects RGCs from apoptosis, and JNK inactivation plays an important role in this process.

Taken together, these results demonstrate that JIP1 deficiency protects RGCs from rotenone-induced apoptosis and functional reduction via JNK inhibition. The repression of JIP1 may be a putative therapy for disorders that are characterized by RGC apoptosis and functional loss.

## Discussion

c-Jun N-terminal kinase (JNK)-interacting protein 1 one of the 4 mammalian JIPs, is highly expressed in the brain (Dickens et al., 1997) and localizes to the neurites and synaptic regions of the cerebellar cortex, olfactory bulb, retina and hippocampus (Pellet et al., 2000). Though JIP1 has been shown to play essential roles in mediating axonal transport, including in the mitochondria, synaptic vesicles (Horiuchi et al., 2005), APP-positive vesicles (Fu and Holzbaur, 2013), and autophagosomes (Fu et al., 2014), the role of retinal JIP1 is still unclear. In the current study, we first investigated the effect of JIP1 deficiency in protecting RGCs from rotenone-induced injury. Second, we examined the molecular signaling alterations associated with JIP1 deficiency-related retinal protection.

Some studies have reported that JIP1 is an important regulator of normal axonal development that promotes axonal growth in some types of neurons (Dajas-Bailador et al., 2008, 2014; Deng et al., 2014). However, the JIP1-deficient mice that we used in this study were viable, had a normal life span, did not display defects in RGC development, and were not different from age-matched WT mice in RGC number (Figure 1), GCC thickness (Figure 2), electrophysiological functions (Figure 3) and the expression of some related proteins, such as pJNK, TUJ1, NF200, PSD95, and SYN (Figure 4 and Supplementary Figure S2). These results suggest that under physiological conditions, mammalian JIP1 may act as an alternative adaptor to maintain the number and function of RGCs and that other proteins may compensate for the loss of JIP1. JIP3 also plays complex roles in the axonal transport of various types of cargo (Koushika, 2008; Huang et al., 2011; Sun et al., 2013). JIP1 and JIP3 cooperate and participate in crosstalk during some biological processes (Song and Lee, 2005; Sun et al., 2017). The transgenic expression of JIP1 in JIP3-null mice partially rescued the axon guidance defects, indicating that there is some functional compensation between JIP1 and JIP3 (Ha et al., 2005). Therefore, we speculate that JIP1 KO mice exhibit no developmental defects due to compensation by JIP3 or other adaptors. Further research is needed to identify the specific cause of this redundancy.

It has been reported that direct intravitreal injection of rotenone in mice represents an acute injury model, in which the rapid loss of RGCs is induced as early as 1 h after injection (Zhang et al., 2002). Researchers have observed the loss of RGCs for 7 days postinjection, with the largest reduction occurring at 24 h postinjection (Heitz et al., 2012; Chadderton et al., 2013), which is consistent with our findings. We also showed that the RGC amounts and PhNR amplitudes of the rotenone-treated eyes were significantly lower than those of the noninjected eyes at 24 h, 3 days, and 7 days after rotenone-injection. The RGC numbers and PhNR amplitudes were stable in WT and JIP1 KO mice, and no significant difference was found at 24 h, 3 days, and 7 days after rotenone-injection. Furthermore, JIP1 KO mice had more RGCs and better RGC function than WT mice at 24 h, 3 days, 7 days after rotenone treatment (Supplementary Figure S3). These results suggest that JIP1 deficiency protects RGCs from loss and dysfunction in an intravitreal injection of rotenone model, and that these effects may last at least 7 days, a long enough period for an acute injury model. In our mouse studies, we focused on a time point of 24 h after intravitreal injection and found that JIP1 deficiency contributed to a greater percentage of surviving RGCs (Figure 1), a thicker RCC (Figure 2), and a significant reduction in the electrophysiological functional loss of RGCs (Figure 3) after intravitreal injection of rotenone compared with those of WT mice. These results demonstrate the protective effects of JIP1 deficiency in a rotenone-induced injury model *in vivo*. Intravitreal injection of rotenone represents a chemically-induced murine model of LHON, which is the most common primary mitochondrial disorder (Yu-Wai-Man et al., 2009). Visual loss occurs in 50% of males and 10% of females with this disorder (Mansergh et al., 2014). Our current study reveals a JIP1 deficiency-related RGC protection mechanism, which may shed light on mitochondrial neurological defects such as LHON.

We also noticed JIP1 expression in the soma and tips of extended neurites in RGCs without any treatment, while most of the JIP1 was located in the soma surrounding the nucleus after rotenone treatment (Figure 5). As previously reported, JNK is mostly located in soma, and when it is activated by a variety of environmental stresses, JNK can translocate to the nucleus and regulate biological process (Leppä and Bohmann, 1999; Kyriakis and Avruch, 2001). In addition, in the case of amyloid β-protein precursor (APP) translocation, for instance, enhanced fast velocity (EFV) is mediated by JIP1 in the interaction of the APP cytoplasmic region with kinesin-1, suggesting these interactions could contribute to EFV generation by inducing a conformational change in the cytoplasmic region (Chiba et al., 2014). Consistent with this possibility, the cytoplasmic region of JIP1 can dynamically alter its conformation. Therefore, we speculate that when the RGCs were injured with rotenone, JIP1 cargo would move from the neurites to the soma. This conformational change in localization was essential for the biological function of JIP1 and involves activating JNK, which further contributes to stress-related apoptosis.

The JIP1 scaffold protein is selectively required for JNK activation in response to specific stimuli. JIP1 mediates JNK activation during the neuronal response to excitotoxin (Whitmarsh et al., 2001). Its activation occurs in many neurodegenerative diseases (Zhu et al., 2001; Hunot et al., 2004; Philpott and Facci, 2008; Miller et al., 2017). We analyzed JNK activity, as determined by phospho-JNK levels, and found that after rotenone injury, the levels of pJNK in the retina and primary RGCs were notably higher than those in the non-treated groups and vehicle-treated groups in both WT and JIP1 KO genotype mice. The level of pJNK in JIP1 KO mice was significantly lower than that in WT mice after rotenone injury (Figures 6, 7). The physiologic role of JIP1 in JNK activation was supported by evidence that JIP1 KO mice lack the ability to elicit anoxic and excitotoxic stress-induced activation of JNK in hippocampal neurons (Whitmarsh et al., 2001), further corroborating our current results showing that JIP1 deficiency retards the sequential kinase activation of the JNK signaling pathway. To confirm the pivotal role of JIP1 in JNK activation and its proapoptotic effect, the caspase-3/cleaved caspase-3 cascades were examined. Caspase-3 is a critical inducer of apoptosis, and the activation of caspase-3 requires the proteolytic processing of its inactive zymogen into activated p17 and p12 fragments (Nicholson et al., 1995). We found that the activation of caspase-3 was lower after rotenone treatment in JIP1-deficient mice than the WT group both *in vitro* and *in vivo* (Figures 6, 7). These results indicate that JIP1 deficiency protects RGCs from apoptosis dependent on the JNK/Caspase-3 signaling pathway.

The identification of signaling molecules downstream of JNK is an important step in understanding the molecular degeneration cascade triggered by rotenone injury. JNKs can phosphorylate a number of substrates, including transcription factors, such as c-Jun, Elk-1, p53, c-Myc, Stat3, and non-transcription factors, such as the Bcl family proteins involved in apoptosis (Bcl-2, Bcl-xL) (Philpott and Facci, 2008), and several of these proteins are important for RGC death after injury (Huang et al., 2005; Lingor et al., 2005; Eichler et al., 2017; Syc-Mazurek et al., 2017). Further research is needed to identify the downstream targets of JNK, which may help to clarify the precise mechanism by which JIP1 leads to JNK activation and subsequent RGC apoptosis and axonal degeneration. In addition, rescue experiments are needed to investigate the unique role of the JIP1-pJNK-caspase-3 axis in order to determine the mechanisms of RGC protection by JIP1 deficiency.

Overall, the results of this study suggest that under developmental and physiological conditions, mammalian JIP1 is not essential for maintaining the number and function of RGCs. JIP1 deficiency is effective in protecting RGCs from apoptosis in a rotenone-induced injured model both *in vitro* and *in vivo*. Agents that target the function of JIP1 may show promise as treatments for LHON or other disorders characterized by RGC apoptosis and functional loss.

## Materials and Methods

### Mice

Mice carrying null alleles for JIP1 were provided by Prof. Yi Rao. And they were backcrossed onto C57BL/6J mice for more than 10 generations and were then intercrossed. A PCR assay was performed using 5′-CGCGGTCTCAGGTGAGCAA-3′ as the common primer, 5′-CTGACTAGGCCTGTAAGAC-3′ as the WT reverse primer, and 5′-CTCCAGACTGCCTTGGGAAAA-3′ as the mutant reverse primer to amplify a 270 bp band for the JIP1 KO allele and a 540 bp band for the WT allele. Mice were fed chow and water *ad libitum* and housed with a 12 h light/dark cycle. Specific-pathogen free (SPF) adult male WT and JIP1 KO mice aged 8–10 weeks (18–24 g) were used. 0.5 μL of rotenone (31.18 mM) was intravitreal injected to induce an injured model (Zhang et al., 2002). The intravitreal injection of 0.5 μL of vehicle (DMSO) was used to rule out the effect of the solvent. Mice without treatment were used as negative control to observe the changes induced by JIP1 KO. The retinas were isolated 24 h after treatment and processed for anatomical and protein studies (Zhang et al., 2002). All animal-related procedures in this study were performed in strict accordance with the Army Medical Center of PLA, Army Medical University (AMU) guidelines for the use of experimental animals. The Animal Ethics Committee of AMU approved all experimental procedures used in the present study.

### RNA Analysis

Total RNA was extracted from the retinas of mice with the indicated genotypes using a PureLink RNA Mini Kit (Ambion) and then reverse-transcribed into cDNA with PrimeScript RT Master Mix (Takara). TaqMan assays were used to quantify JIP1, and the amount of mRNA was measured by quantitative reverse transcription-PCR (RT-PCR) using the primers GGCCTACCACGCTCAACCTT and ACACACGGTCCTGCCAACTG. The relative mRNA expression level was normalized to the amount of β-actin RNA in each sample.

### Culture of Primary RGCs

Primary RGCs were cultured using methods that were described previously (Zhou et al., 2018). Briefly, retinas were isolated from WT and JIP1 KO mice within 48 h of birth, purified by using an RGC-specific Thy-1 antibody (LS-C14009, LifeSpan, United States) and cultured in serum-free neurobasal medium with B27 supplements (×1, Gibco, United States) at 37°C with 5% CO_2_ for 6 days. Then, the cells were treated with 0.5 μg/mL rotenone or vehicle in normal medium. Twelve hours later, the cells were fixed or collected for further analysis.

### SD-OCT

A SD-OCT system (Heidelberg Engineering, Heidelberg, Germany) was used for retinal scanning, before and after treatment with vehicle or rotenone as previously described (Zhou et al., 2018). Mice were anesthetized by intraperitoneal injection of pentobarbital sodium (40 mg/kg), and tropicamide (1%; Alcon-Cusi, Barcelona, Spain) was dropped in the eyes to induce pupil dilation. The mice were placed on a mouse holder. Retinas were scanned with a raster pattern of 31 equally spaced horizontal B-scans spanning the central retina. The GCC thickness (ganglion cell complex, a combination of the retinal nerve fiber layer, ganglion cell layer and inner plexiform layer) was calculated in an area 1500 μm away from the ON disk. Six individual eyes from either group were analyzed separately.

### Electroretinography

A full-field flash ERG was recorded using an Espion system (Diagnosys, MA, United States) based on previous literature (Chrysostomou and Crowston, 2013; Frishman et al., 2018). Animals were subjected to light adaptation for at least 15 min and then anesthetized as previously described. Corneal anesthesia and mydriasis were achieved by the topical application of oxybuprocaine (0.4%) and tropicamide (0.5%). Electrical signals were recorded with a platinum wire loop electrode that contacted the cornea, and a gold pellet was placed in the mouth to serve as a common reference. A subdermal needle electrode was inserted at the base of the tail and acted as a ground. Light energies were calibrated as luminance energy units in candela seconds per meter squared (cd.s/m^2^). photopic responses to different stimulus strengths were recorded in a rod-saturating green background. At each intensity, 25 flashes were averaged, with an interstimulus interval of 3000 ms. The amplitudes of the PhNR were measured from baseline to the PhNR trough. Six individual eyes were obtained from each group and analyzed separately.

### Retinal Histology and Cell Counts

Animals were sacrificed by an overdose inhalation of CO_2_. As previously described (Lin et al., 2012), after 0.9% saline perfusion, eyes were fixed in 4% paraformaldehyde (PFA) for 1 h at room temperature (RT). The retinas were dissected from the optic cups, cut into four quadrants, and permeabilized in 3% Triton X100 solution overnight at 4°C. Then, the retinas were blocked using 3% goat serum for 2 h at RT and incubated for 3 days at 4°C in primary antibody for βIII tubulin (Table 1). Retinal wholemounts were washed and incubated at 4°C in fluorescently labeled secondary antibody overnight at 4°C. Images were captured by an SP-8 confocal microscope (Leica, Germany). The retinas were examined for RGCs at a distance of 1 or 2 mm from the center of the ONH to examine the proximal (*p*) and distal (*d*) regions, respectively. βIII Tubulin-positive cells were counted in the proximal or distal regions from four fluorescent images (one image per retinal quadrant) per retina. RGC densities were measured in 12 distinct images, and the number was averaged to estimate the overall RGC survival.

**TABLE 1:** List of antibodies used for western blot (WB), immunohistochemistry (IHC) and immunocytochemistry (ICC).

**Antibody**	**Sources**	**Catalog Number**	**Dilution**	
			
			**WB**	**IHC/ICC**
**Primary antibodies**				
Mouse anti-JIP1	Santa Cruz Biotechnology, San Diego, CA, United States	SC25267	1:500	1:500
Mouse anti-Brn3a	Merck Millipore, Watford, United Kingdom	MAB1585		1:20
Rabbit anti-βIII tubulin	Abcam, Cambridge, United Kingdom	ab18207		1:1000
Mouse anti-βIII tubulin	Abcam, Cambridge, United Kingdom	ab78078	1:500	1:500
Rabbit anti-JNK	Abcam, Cambridge, United Kingdom	ab179461	1:1000	1:250
Rabbit anti-phospho-JNK	Abcam, Cambridge, United Kingdom	ab124956	1:1000	1:100
Rabbit anti-caspase-3	Cell Signaling Technology, Danvers, MA, United States	9662	1:1000	
Rabbit anti-cleaved caspase-3	Cell Signaling Technology, Danvers, MA, United States	9661	1:1000	
Rabbit anti-GAPDH	Abcam, Cambridge, United Kingdom	ab9485	1:5000	
Rabbit anti-NF200	Abcam, Cambridge, United Kingdom	ab8135	1:1000	1:1000
Rabbit anti-PSD95	Abcam, Cambridge, United Kingdom	ab18258	1:1000	1:500
Rabbit anti-SYN	Abcam, Cambridge, United Kingdom	ab14692	1:1000	1:200
**Secondary antibodies**				
HRP-labeled anti-mouse IgG	Thermo Fisher Scientific, Waltham, MA, United States	31430	1:5000	
HRP-labeled anti-rabbit IgG	Thermo Fisher Scientific, Waltham, MA, United States	31460	1:5000	
Alexa488 anti-mouse IgG	Abcam, Cambridge, United Kingdom	ab150113		1:500
Alexa488 anti-rabbit IgG	Abcam, Cambridge, United Kingdom	ab150077		1:500
Alexa594 anti-mouse IgG	Abcam, Cambridge, United Kingdom	ab150116		1:500
Alexa594 anti-rabbit IgG	Abcam, Cambridge, United Kingdom	ab150080		1:500

### Immunohistochemistry of Retinal Sections and Primary RGCs

Whole eyes were removed and fixed for 2 h in 4% PFA, dehydrated in a sucrose solution, embedded in OCT Tissue Tek Medium, cut into 10-μm-thick sections using a cryostat (Bright Instruments, Huntingdon, United Kingdom) and adhered onto anti-slip slides, as described previously (Vigneswara et al., 2015). Cultured primary RGCs were fixed in 4% PFA for 15 min at RT. Retinal sections and fixed cultured primary RGCs were washed in PBS, permeabilized and blocked in 0.1% Triton X100 and 3% bovine serum albumin in PBS for 30 min at RT. Then, they were incubated with appropriate primary antibodies (Table 1) overnight at 4°C. After these steps, the samples were washed in PBS and incubated with the appropriate fluorescent secondary antibodies (Table 1) for 1 h at RT. They were mounted after DAPI staining. An SP-8 confocal microscope was used to capture images.

### TUNEL Assay

Apoptosis of retinas and primary RGCs was evaluated by TUNEL assays (Sigma, Shanghai, China). Retinal Cryo-sections and primary RGCs were fixed by 4% PFA for 15 min at RT. After washed in PBS, the samples were permeabilized in 0.1% citrate/Triton buffer for 15 min at RT, and incubated with the TUNEL reaction mixture for 1 h at 37°C in a dark and humidified box. Nuclei were stained using DAPI for 5 min. Samples were visualized using an SP-8 confocal microscope. The number of TUNEL-positive cells in the GCL or primary RGCs was counted randomly.

### Protein Extraction and Western Blot Analysis

Protein extraction and Western Blotting were performed, as previously described (Lin et al., 2012). Briefly, retinal tissues obtained following *in vivo* experiments or primary cultured RGCs *in vitro* were lysed with ice-cold lysis buffer, which consisted RIPA and a protease inhibitor cocktail (Roche Diagnostics, IA, United States). Then, the lysates were collected and clarified by centrifugation. Twenty micrograms of total protein extract was resolved on 10% SDS-polyacrylamide gels, transferred to polyvinylidene fluoride membranes (Millipore Corporation, Bedford, MA, United States) and probed with relevant primary antibodies (Table 1), followed by incubation with corresponding secondary antibodies. Images were captured using a gel imaging system (Aplegen, San Francisco, CA, United States). The phospho-JNK and cleaved-caspase-3 levels were normalized to that of JNK and pro-caspase-3, respectively. The normalized ratio for the WT with nontreatment group was set as 1.

## Statistical analysis

SPSS 18.0 was used to perform all statistical analyses. Graphs present the mean and standard error of the mean (SEM). Experiments comparing differences were analyzed using one-way ANOVA followed by the LSD test for group comparisons. *P*-values <0.05 were considered statistically significant.

## Data Availability Statement

All datasets generated for this study are included in the manuscript/Supplementary Files.

## Ethics Statement

The animal study was reviewed and approved by Animal Ethics Committee of AMU.

## Author Contributions

SL designed the experiments, supervised the project, interpreted the data and modified the manuscript. JY provided the funding and discussed the study. WL performed the experiments, analyzed the data and wrote the manuscript. XL and XC helped in Western Blotting and immunostaining. JZ helped in intravitreal injection. LL and QH helped in SD-OCT measurement. JZ helped in raising animals. JunY helped in study discussion. All authors read and approved the final manuscript.

## Conflict of Interest

The authors declare that the research was conducted in the absence of any commercial or financial relationships that could be construed as a potential conflict of interest.

## Supplementary Material

FIGURE S1 Validation of JIP1-deficient mice. **(A)** Genomic DNA was prepared from wild-type (WT) (JIP1+/+), heterozygous (JIP1±), and homozygous (JIP1-/-) mice. Genotype analysis was performed by PCR. **(B)** The expression of JIP1 mRNA in the retina of WT and JIP1 KO mice was measured by quantitative RT-PCR analysis. The relative mRNA expression was calculated by normalizing the mRNA levels to the β-actin mRNA level in each sample (^∗^*P* < 0.05, *n* = 3). **(C)** Retinal tissues from WT and JIP1 KO mice were examined by immunoblot analysis using antibodies against JIP1 and GAPDH.

FIGURE S2 The expression of proteins relates to RGCs under physiological conditions. **(A)** Immunostaining of TUJ1, NF200, PSD95, SYN (green) and DAPI (blue) in retinal sections from WT and JIP1 KO mice (scale bar = 50 μm). **(B)** Representative images of Western blot results of TUJ1, NF200, PSD95, SYN and the loading control GAPDH in the retinas of WT and JIP1 KO mice. **(C)** Quantification of the Western blot results of TUJ1, NF200, PSD95, and SYN in WT and JIP1 KO mice (^∗^*P* < 0.05, *n* = 3). TUJ1, neuronal class III β-tubulin; NF200, neurofilament; PSD95, postsynaptic density 95; SYN, synaptophysin.

FIGURE S3 JIP1 deficiency protects RGCs from loss and dysfunction for at least 7 days. **(A)** RGCs were immunostained using a TUJ1 antibody in the distal regions of retinal wholemounts from WT and JIP1 KO mice in the non-injection (NI) group, and at 24 h, 3 days, and 7 days after rotenone-injection (scale bar = 50 μm). **(B)** Quantification of TUJ1-positive cells in the distal regions (^∗^*P* < 0.05, *n* = 12 images). **(C)** Representative traces demonstrating the PhNR components (arrows) that were recorded from a mouse in the NI group, and at 24 h, 3 days, and 7 days after rotenone-injection with a stimulus strength of 41.68 cd.s/m^2^. **(D)** Quantification of PhNR amplitudes with a stimulus strength of 41.68 cd.s/m^2^ (^∗^*P* < 0.05, *n* = 6).
