# Supplementary figures and images for: JIP1 Deficiency Protects Retinal Ganglion Cells From Apoptosis in a Rotenone-Induced Injury Model
Source: Front Cell Dev Biol. 2019 Oct 15;7:225. doi: 10.3389/fcell.2019.00225 (PMC6804425; doi:10.3389/fcell.2019.00225)

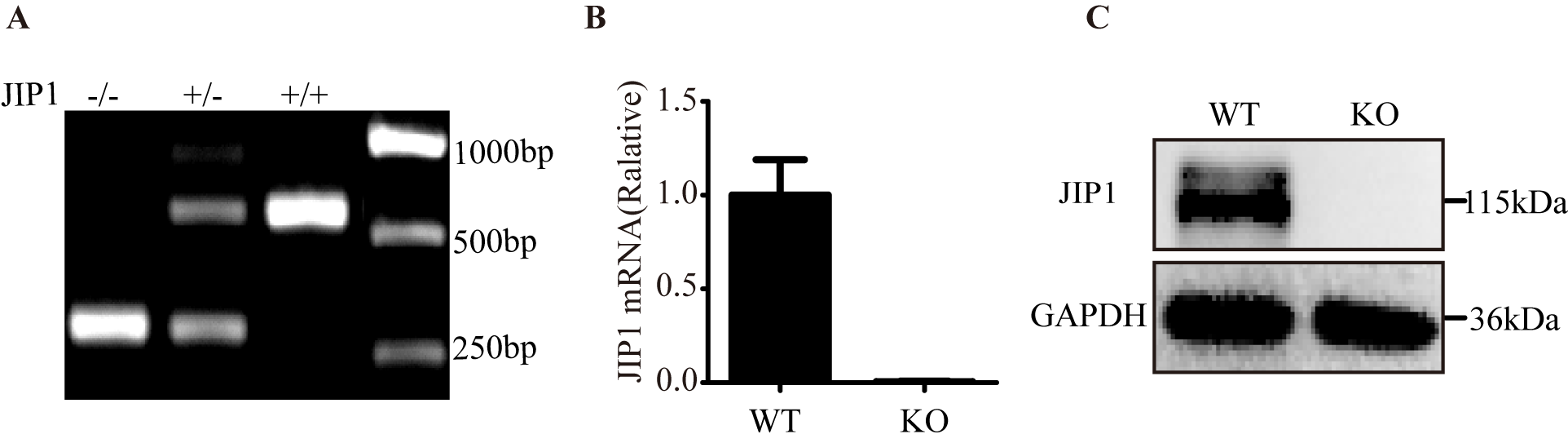

Supplement: FIGURE S1 — Validation of JIP1-deficient mice. (A) Genomic DNA was prepared from wild-type (WT) (JIP1+/+), heterozygous (JIP1±), and homozygous (JIP1-/-) mice. Genotype analysis was performed by PCR. (B) The expression of JIP1 mRNA in the retina of WT and JIP1 KO mice was measured by quantitative RT-PCR analysis. The relative mRNA expression was calculated by normalizing the mRNA levels to the β-actin mRNA level in each sample (∗P < 0.05, n = 3). (C) Retinal tissues from WT and JIP1 KO mice were examined by immunoblot analysis using antibodies against JIP1 and GAPDH. [file Image_1.TIF]

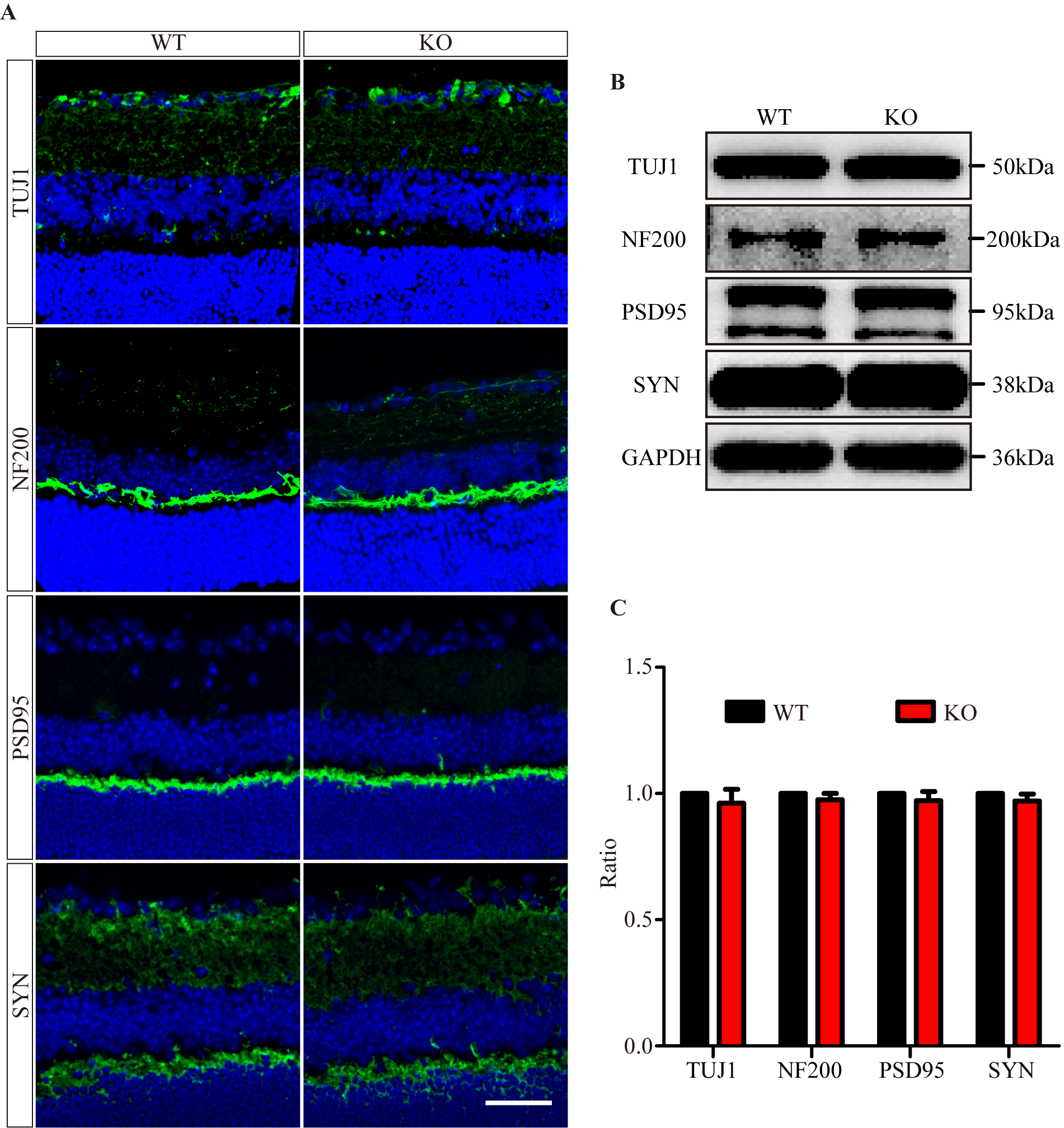

Supplement: FIGURE S2 — The expression of proteins relates to RGCs under physiological conditions. (A) Immunostaining of TUJ1, NF200, PSD95, SYN (green) and DAPI (blue) in retinal sections from WT and JIP1 KO mice (scale bar = 50 μm). (B) Representative images of Western blot results of TUJ1, NF200, PSD95, SYN and the loading control GAPDH in the retinas of WT and JIP1 KO mice. (C) Quantification of the Western blot results of TUJ1, NF200, PSD95, and SYN in WT and JIP1 KO mice (∗P < 0.05, n = 3). TUJ1, neuronal class III β-tubulin; NF200, neurofilament; PSD95, postsynaptic density 95; SYN, synaptophysin. [file Image_2.TIF]

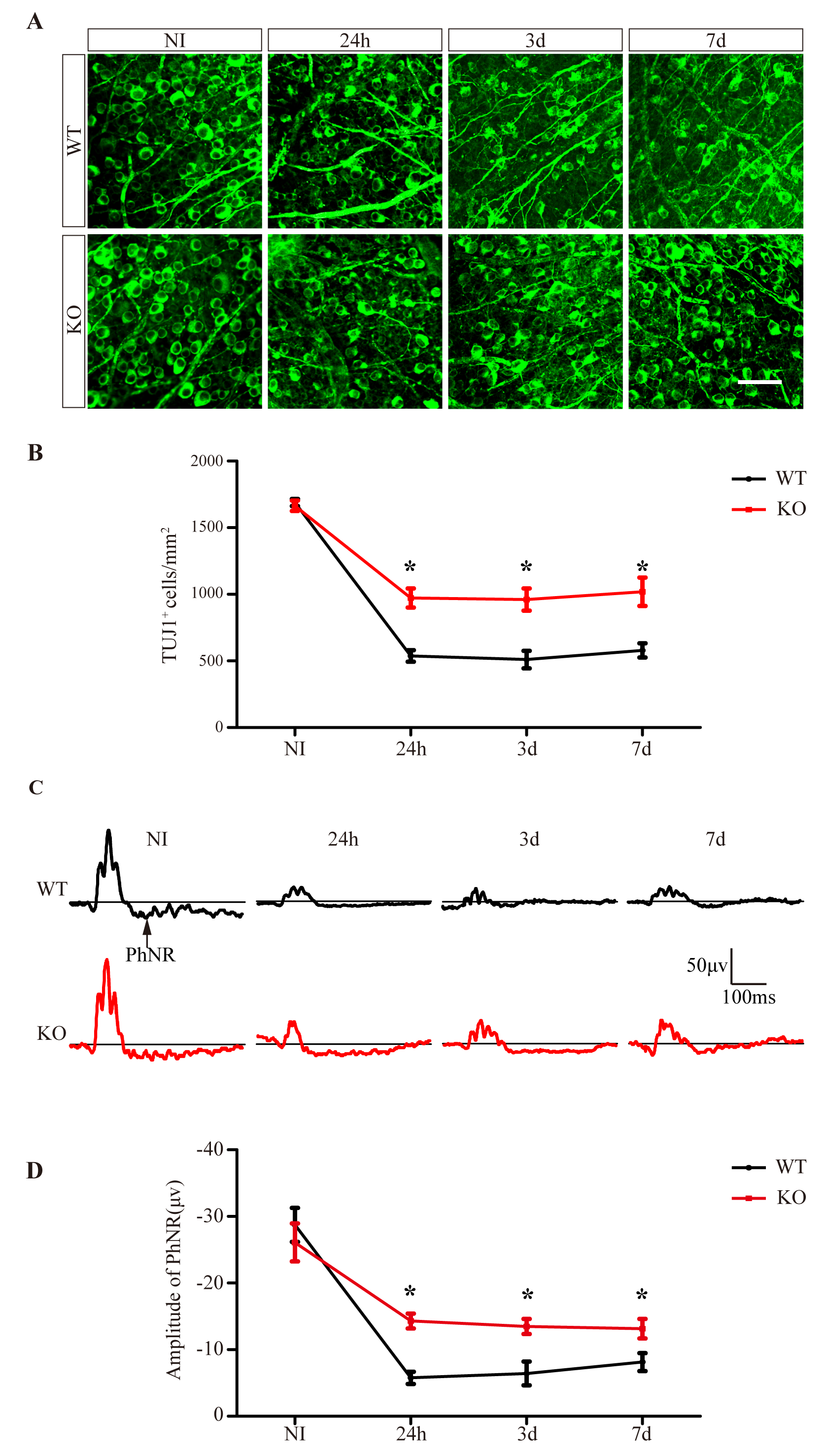

Supplement: FIGURE S3 — JIP1 deficiency protects RGCs from loss and dysfunction for at least 7 days. (A) RGCs were immunostained using a TUJ1 antibody in the distal regions of retinal wholemounts from WT and JIP1 KO mice in the non-injection (NI) group, and at 24 h, 3 days, and 7 days after rotenone-injection (scale bar = 50 μm). (B) Quantification of TUJ1-positive cells in the distal regions (∗P < 0.05, n = 12 images). (C) Representative traces demonstrating the PhNR components (arrows) that were recorded from a mouse in the NI group, and at 24 h, 3 days, and 7 days after rotenone-injection with a stimulus strength of 41.68 cd.s/m2. (D) Quantification of PhNR amplitudes with a stimulus strength of 41.68 cd.s/m2 (∗P < 0.05, n = 6). [file Image_3.TIF]
